# Water use of *Prosopis juliflora* and its impacts on catchment water budget and rural livelihoods in Afar Region, Ethiopia

**DOI:** 10.1038/s41598-021-81776-6

**Published:** 2021-01-29

**Authors:** Hailu Shiferaw, Tena Alamirew, Sebinasi Dzikiti, Woldeamlak Bewket, Gete Zeleke, Urs Schaffner

**Affiliations:** 1 Department of Geography and Environmental Studies, Addis Ababa University, P.O. Box 1176, Addis Ababa, Ethiopia; 2 Water and Land Resource Centre, Addis Ababa University, P.O. Box 3880, Addis Ababa, Ethiopia; 3 CSIR Smart Places Cluster, 11 Jan Cilliers Street, Stellenbosch, 7599 South Africa; 4 CABI, Rue des Grillons 1, 2800 Delémont, Switzerland; 5 Department of Horticultural Science, University of Stellenbosch, P. Bag X1 Matieland, Stellenbosch, 7602 South Africa

**Keywords:** Hydrology, Ecology, Ecophysiology, Invasive species

## Abstract

Dense impenetrable thickets of invasive trees and shrubs compete with other water users and thus disrupt ecosystem functioning and services. This study assessed water use by the evergreen *Prosopis juliflora,* one of the dominant invasive tree species in semi-arid and arid ecosystems in the tropical regions of Eastern Africa. The objectives of the study were to (1) analyze the seasonal water use patterns of *P. juliflora* in various locations in Afar Region, Ethiopia, (2) up-scale the water use from individual tree transpiration and stand evapotranspiration (ET) to the entire invaded area, and 3) estimate the monetary value of water lost due to the invasion. The sap flow rates of individual *P. juliflora* trees were measured using the heat ratio method while stand ET was quantified using the eddy covariance method. Transpiration by individual trees ranged from 1–36 L/day, with an average of 7 L of water per tree per day. The daily average transpiration of a *Prosopis* tree was about 3.4 (± 0.5) mm and the daily average ET of a dense *Prosopis* stand was about 3.7 (± 1.6) mm. Using a fractional cover map of *P. juliflora* (over an area of 1.18 million ha), water use of *P. juliflora* in Afar Region was estimated to be approximately 3.1–3.3 billion m^3^/yr. This volume of water would be sufficient to irrigate about 460,000 ha of cotton or 330,000 ha of sugar cane, the main crops in the area, which would generate an estimated net benefit of approximately US$ 320 million and US$ 470 million per growing season from cotton and sugarcane, respectively. Hence, *P. juliflora* invasion in the Afar Region has serious impacts on water availability and on the provision of other ecosystem services and ultimately on rural livelihoods.

## Introduction

In the context of climate change and rising demands by a growing human population, water is becoming an increasingly scarce resource^1^. Human societies in arid and semi-arid regions are particularly prone to water scarcity during at least some parts of the year^2^. In those regions, meeting the rising demands for freshwater and protecting or restoring ecosystems so that they can sustainably replenish water stocks will be one of the most difficult and important challenges of this century.

Water is lost through transpiration, interception and evaporation. Evapotranspiration, which can account for more than 90% of the water balance in some catchments, depends on the climatic factors e.g., solar radiation, air temperature, air humidity and wind speed^3–5^. In addition, the loss of water to the atmosphere is also affected by the available soil moisture, vegetation structure and the eco-physiology of the dominant plant species^6,7^. Species traits such as plant physiology (e.g. ever greenness), size and root architecture affect water availability and water use both at the individual plant as well as at the ecosystem level^8,9^. For example, at the watershed level, afforestation of grasslands and shrubland can result in significant reduction of stream flow^7^.

Besides land-use change and land degradation, invasive alien plant species (IAPS) are among the most important drivers of change in vegetation composition and ecosystem functioning in terrestrial ecosystems^10,11^. Due to their invasive character, these plants can become the dominant species both at the local as well as at the landscape scale. Some of the well-known examples of changes in vegetation composition due to plant invasions originate from invasions of grasslands or shrubland by woody IAPS in arid and semi-arid environments, such as in South Africa^9^ and Australia^12^. In a review of the water use by native and invasive plant species, Cavaleri and Sack^13^ and Dzikiti et al^14^ found that individual native and invasive trees tended to have similar sap flow rates, but that invasive-dominated ecosystems were more likely to have higher sap flow rates per unit area than native-dominated ecosystems because invasive species grow in denser stands than natives species and dense stands consume more water than sparsely stocked stands.

**Figure 1:**
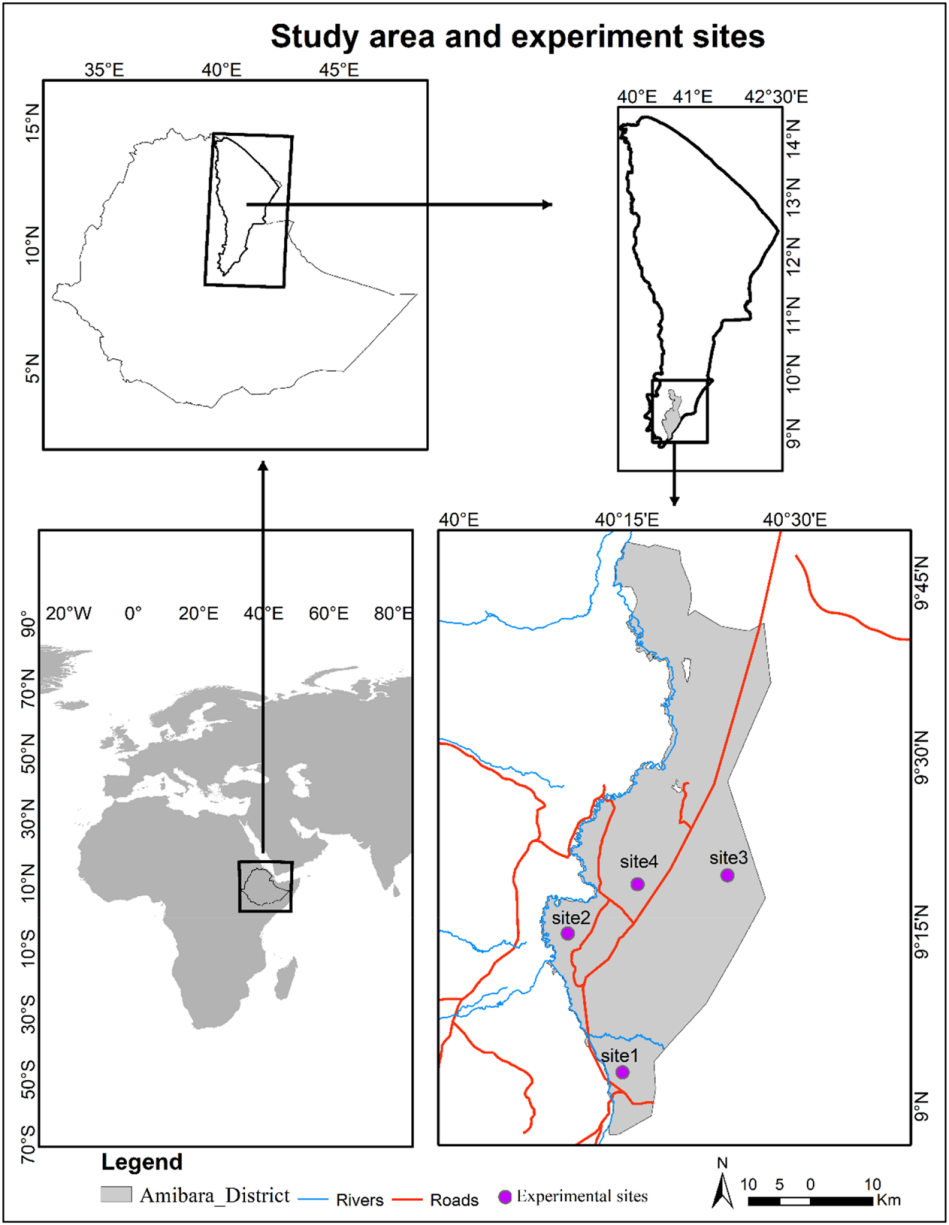
Location of study area and experimental sites in the Afar Region, Ethiopia

Species of the genus *Prosopis*, and their hybrids, are among the most aggressive IAPS worldwide, particularly in semi-arid and arid regions^15^. *Prosopis* species, native to Central and South America were introduced to different parts of the world, including Africa, India and Australia, to provide shade, shelter, fodder, fuel wood and timber, and to increase soil stability in degraded ecosystems^16,17^. However, several of the planted *Prosopis* species, including *Prosopis juliflora* (Swartz D.C), have become invasive, thereby displacing native vegetation, reducing biodiversity and negatively affecting rural livelihoods^15,18–20^. Moreover, *Prosopis* species have been reported to be highly water-consuming plant species^14,21^. Studies conducted in South Africa revealed that the invasive *Prosopis* tree species and hybrids significantly impact groundwater levels^22,23^, due to the very deep taproots. Groundwater abstraction (i.e. the process of extracting ground water) by *Prosopis sp*. affects both surface runoff, groundwater recharge and evaporation losses^9^. So far, studies on the eco-hydrology of *Prosopis* have been conducted in its native range in the Southern Arizona, USA^24,25^ (mainly *Prosopis glandulosa* Torr.) and in the introduced range in South Africa^14,22^ (complex of *Prosopis* species and hybrids) where *Prosopis* trees shed their leaves during the cold season. However, no study has investigated water consumption of the evergreen *P. juliflora*, the dominant invasive *Prosopis* species in semi-arid and arid ecosystems in the tropical regions of Eastern Africa. The evergreen leaf habit of *P. juliflora* may lead to year-round groundwater abstraction by this species. Thus, the overall water consumption of the evergreen *P. juliflora* is likely to be higher than that of leaf-shedding *Prosopis* species. In this paper, we quantified the diurnal and seasonal water use of *P. juliflora* trees in the flood plains and the adjacent drylands of the semi-arid Afar Region, Ethiopia (Fig. 1). We also assessed evapotranspiration of a dense *P. juliflora* stand in the floodplains. To upscale water consumption by *P. juliflora* to the regional level, we combined the stand level water use measurements with the fractional vegetation cover of *P. juliflora* in the Afar Region that was mapped and published by Shiferaw et al^26^. Finally, we estimated the annual costs of water use by *P. juliflora* at the regional level.

## Materials and methods

### Study area

Water consumption of *P. juliflora* (hereafter *Prosopis*) was measured in the Amibara District (Fig. 1) of the Afar Region, Ethiopia (9.16° to 9.21° N and 40.08° to 40.12° E at 740 m a.s.l.). The study area is located in the Awash River Basin and includes both the floodplains of the Awash River and the adjacent dryland. Although relatively water scarce, the Awash River Basin is the most developed and utilized river basin in Ethiopia^27^. The human and livestock population in this basin are estimated at 18.6 and 34.4 million, respectively, and nearly 70% of large irrigation schemes in Ethiopia are located in the Awash Basin^28^. The mean annual river flow/discharge of the basin at the terminal Lake Abe is estimated at about 4.6 billion m^3^ of water although exposed to evaporation^27^. Water scarcity, particularly in the lower parts of the river, is the major limiting factor for irrigation development, particularly during the low flow season^27^.

The Afar Region has a mean annual rainfall of about 560 mm^29^. The region is the hottest part of Ethiopia, with a mean annual temperature of 31 °C. The mean maximum temperature reaches up to 41 °C in June, and the mean minimum temperature ranges from 21 to 22 °C between November and December^30^. The biome can be described as semi-arid to semi-desert. The natural vegetation consists of scattered dry shrubs, woodland comprising different *Vachellia* (*Acacia*) species, bushland, grassland and wooded grassland^18^. The area has different soil types, including silt fertile soils, sandy soils, heavy clays and rocky outcrops, and a wide range of altitudes ranging from 175 m below sea level to 2,992 m a.s.l. Shiferaw et al.^30^ found that *Prosopis* has primarily invaded areas ranging from rangelands to farmland. Main sources of livelihood are pastoralism and some agro-pastoralism around small rural towns^30^. The main crops grown in the floodplains of Awash River are cotton and sugarcane.

### Experimental design

To investigate the temporal and spatial variation in water use by *Prosopis* across the landscape, data collection was done in the two most heavily invaded habitat types in the Afar Region. These include the floodplains of Awash River and the adjacent non-riparian drylands. Sap flow monitoring stations were established at two sites in the floodplains of the Awash River and at two sites in the dryland area where soil moisture levels were low (Fig. 1). The study areas were representative of other parts of the Afar Region that are invaded by *Prosopis*.

Site 1 was located near Worer Agricultural Research Center some 200 m away from Awash River and about 30 m from a nearby irrigation canal, and was considered a floodplain site. The area was used for crop production until 2012 after which it was abandoned due to shallow water inducing soil salinity problems. Soon after abandonment, *Prosopis* invaded the area and established dense stands with 100% canopy cover in most places comprising trees up to 6.5 m height. Soil moisture was relatively high at Site 1 due to proximity to the river. The area invaded by *Prosopis* was about 6 ha and the soils were temporarily flooded loam and clay soils. Except for annual grasses in some open spaces, there was no undergrowth vegetation, probably due to the dense *Prosopis* cover.

Site 2 was in the floodplains of a tributary of Awash River and located near Berta locality (Fig. 1). There is a continuous flow of water from a pumped well to less than 10 m from the site where sap flow was measured. The soil is a sandy loam formation, which maintains relatively high moisture content. The *Prosopis* stand was about 3 ha in size, had a closed canopy and comprised trees of more than 5 m height.

Site 3 was on dryland in the former rangeland of Hallaideghe locality (Fig. 1), characterized by sandy loam soil formation. There was no surface water source except from rainfall and seasonal flooding from the West Harerghe highlands. This area is now invaded by *Prosopis* with a closed canopy and tree heights of close to 5 m. The size of the invaded area was about 100 ha.

Site 4 was in the drylands of Berta locality (Fig. 1). The soil is sandy with a high rocky outcrop. The dominant indigenous vegetation around this site consisted of a few *Senegalia senegal* (L.) Britton (or *Acacia senegal* (L.) Willd) and other small shrubs and grasses. The area is now dominated by *Prosopis* stands with a closed canopy with trees reaching up to 4 m height. At this site, a meteorological station was installed next to the sap flow monitoring equipment.

The sap flow data in this study therefore provide insight into how tree water use varied across the landscape including (a) floodplains, (b) dryland areas, and (c) across these two most heavily invaded habitats in the Afar Region. All experimental sites were fenced and protected from animal and human interference. Moreover, safety boxes were also made to protect the equipment from weather and other damages.

### Tree and stand water use measurements

The amount of water used by individual *Prosopis* trees was determined using the heat ratio method (HRM) of monitoring tree sap flow^31^. This technique was selected because it is suitable for measuring low and reverse sap flows which are likely present in desert-adapted species such as *Prosopis*^14,32^. In total, four sap flow stations were established (Sites 1 to 4) with three trees instrumented per station. Trees with different stem diameters were selected to capture the variation in transpiration rates in the study region. Stem diameter of the instrumented trees was measured just below the branching at about 60 cm above the ground at all sites. Each sap flow station comprised a CR1000 data logger and an AM16/32B multiplexer, as specified by Campbell Scientific, Inc., Logan UT, USA. Each system was powered by a 70 Ah (12 V) rechargeable battery using 50 W solar panels. Four sets of heaters applied heat to each tree for 0.5 s every hour through a custom-made relay control module. Moreover, a pair of equally placed (0.5 cm) T-Type thermocouples was installed on either side of the heater to measure the sapwood temperature before and after pulsing the heat. With a precision drilling rig, two 2.0 mm diameter holes were carefully made for the thermocouples to minimize errors due to probe misalignments. Heater holes were about 1.8 mm diameter to ensure a tight fit to facilitate heat transfer to the wood during pulsing.

In this sap flow monitoring technique, the heat pulse velocity (Vh, cm/h) is logarithmically related to the ratio of temperature increases upstream and downstream from a heater (v_1_/v_2_) as shown in Eq. (1), Burgess et al^31^:

1 $$Vh=\left(\frac{k}{x}\right)(\mathrm{ln}\left(\frac{v1}{v2}\right))\times 3600$$

where *Vh* is heat velocity cm/hour, *k* is the thermal diffusivity which was assigned a nominal value of 2.5 × 10^–3^ cm^2^/s for wood, *x* is the distance (cm) between the heater and either temperature probe (~ 0.5 cm), and *v*_*1*_ and *v*_*2*_ are increases in temperature before and after pulsing^31^.

The thermocouples were installed in the sapwood at depths ranging from 0.8 to 1.1 cm under the bark to capture the radial changes in sap velocity. Wounding corrections were applied according to the method described by Swanson and Whitfield^57^. The depth of the sapwood was determined visually as it was possible to distinguish between the sap wood and heartwood boundaries from the changes in the color of the wood. The individual tree sap flow volume in liters per hour were converted to stand level transpiration (in mm per hour) using the approach described by Dzikiti et al^14^ in which the instrumented trees were assigned to a particular stem size class. The stand level transpiration was then calculated as a weighted sum of the transpiration rates by the trees in each stem size class with the proportion of trees in each size class as the weights. The volumetric soil water content in the root-zone of the trees were measured at each site using a single soil water content reflectometer probe (Model CS616: Campbell Scientific, Inc., Logan UT, USA) installed horizontally at a depth of 50 cm. Sap flow and soil moisture data were measured for 15 months (from November 2016 to January 2018) while evapotranspiration was measured for 11 months (from January to November 2017).

To study the dynamics of total actual evapotranspiration (ETa) from *Prosopis* stands, an open path eddy covariance (EC) system was installed on Site 1 and data were collected for 11 months from January to November 2017 as it was not possible to continue measuring for more periods due to equipment limitations. The EC was borrowed from Addis Ababa University only for one year so ETa from the other sites could not be measured. The EC system was the IRGASON system which comprised a sonic anemometer (Model: CSAT3A Campbell Scientific Inc., Logan UT, USA) that measured the wind speed in 3-D at 10 Hz frequency. The H_2_O/CO_2_ concentrations of the atmosphere were measured using an Infrared Gas Analyzers (Model: EC150, Campbell Scientific, Inc., Logan UT, USA). The collected data was stored by a data logger (Model: CR3000: Campbell Scientific, Inc., Logan UT, USA) on a Compact Flash card module (NL115 or CFM100). To quantify the changes in the energy balance of the study site, two other components of the surface energy balance were measured. These include the net radiation, which was measured using a single component net radiometer (Model: NR-LITE2: Manufacturer: Kipp & Zonnen, Delft, The Netherlands) that was mounted at the top of the tower (~ 7.5 m above the ground). The IRGASON sensor was installed outside the surface roughness layer of the canopy at an average height of about one meter above the *Prosopis* tree canopy. This ensured a uniform fetch around the tower with a flux foot print of about 100 m radius.

Air temperature and humidity were measured at high frequency using a temperature and humidity Probe (Model HMP155A-L, Campbell Scientific, 2013). The high frequency data were further corrected for 1) lack of sensor levelness (coordinate rotation), 2) sensor time lags, and 3) fluctuations in the air density using the EddyPro version 6.0 software (Li-COR, Nebraska, USA). Sensor separation corrections were not necessary as the IRGA and sonic are a single unit.

Allometric characteristics are one of the major biological factors affecting the eco-physiology of plant species. For example, sapwood area is usually correlated with stem diameter^14^. The sapwood area estimated from the stem diameter measurements was used to calculate the sap flow volumes from the sap velocity measured by the HRM system.

### Weather and soil water dynamics

To measure solar irradiance, precipitation, air temperature, relative humidity and air pressure, an automatic weather station was set up at Site 4, which was located within 7 km from the other three sites. The solar radiation sensor was installed on a horizontal leveling fixture mounted on a south facing cross bar to avoid self-shading errors. A wind sentry was used to measure the wind speed and direction (Model 03,001, R.M. Young; Campbell Scientific, Inc., Logan UT, USA). Rainfall was monitored using a tipping bucket rain gauge (Model TE525-L, Campbell Scientific, Inc., Logan UT, USA). The weather station comprised an Em50 (a 5-channel data logger) and ECH_2_O utility software from Decagon, USA.

Wind speed was obtained from the weather station located at Worer Agricultural Research Center, which was about 500 m away from Site 1. The weather station had a temperature and humidity probe (Model CS500, Vaisala, Finland) installed at a height of about 2.0 m above ground and the station also measured wind speed using a cup or rotational anemometer installed at 2 m high.

The energy transferred into and out of the ground was measured using clusters of soil heat flux plates (Model: HFP01SC-L, Delft, The Netherlands), while soil temperature was recorded using soil averaging thermocouples (Model: TCAV-L: Campbell Scientific, Inc., Logan UT, USA). The soil heat flux plates were installed at 8 cm depth and the soil averaging and soil moisture data measured with the soil water content reflectometers (Model: CS616-L: Campbell Scientific, Inc., Logan UT, USA) were used to correct the soil heat flux for the energy stored by the soil layer above them. At all sites, the sensors were connected to a data logger (Model CR1000, Campbell Scientific, Inc., Logan UT, USA) programmed with a scan interval of 90 s, and data were stored at hourly intervals over the 11 months study period. All data were downloaded every 21 days from data loggers.

### Drivers of water use by the invasive Prosopis

To identify the main drivers of water use by *Prosopis* invasions in the Awash River basin of the Afar Region correlations were sought between the various water use variables (transpiration and evapotranspiration) as dependent variables and microclimate factors, i.e. solar radiation, wind speed, vapor pressure deficit of the air (VPD), soil moisture, and ET_0_ as explanatory variables.

### Upscaling Prosopis water use moderation to the Afar Regional level

To upscale the *Prosopis* transpiration and ET from the individual study sites to the regional scale, a regression equation was developed using the fractional vegetation cover information mapped by Shiferaw et al^26^. This mapping quantified the *Prosopis* distribution and cover in the Afar Region at a 15 × 15 m spatial resolution based on explanatory variables including Landsat panchromatic images, other biophysical parameters and field observations. The fractional cover map was generated using a robust modelling approach, Random Forest Algorithm, with a large amount of field observations (> 3000 plots) and seventeen explanatory variables^33^. Then, we estimated the amount of water used by *Prosopis* stands at each of the four sites in mm/day per pixel with 100% cover. This was extrapolated to all fractional cover levels per pixel indicated in the fractional cover map for *Prosopis* in the study area. Moreover, canopy cover from the experimental plots of *Prosopis* trees was estimated. Then, the relationship between sap flow and fractional cover as well as between ET and fractional cover (F_ci_) over the invaded area was developed as shown in Eq. (2):

2 $${\text{f }}\left( {\text{x}} \right) = \sum ({\text{Fci}}({\text{Wi}})S)$$

where *f(x)* is either total water use (sap flow) or total ET in mm/day over the whole study area; *Fc*_*i*_ is fractional cover at pixel level _i_, *W*_*i*_ is water use either from sap flow or stand ET at 100% canopy cover, and *S* is a pixel size of 225 m^2^.

Finally, we estimated the financial costs incurred from the loss of water through *Prosopis* transpiration and ET. This was done by taking the water charge of payment for ecosystems services by investors to Awash Basin Organization which was set at US$ 0.00015 per m^3^ according to Ayana et al^34^. Also, we estimated the market price and the net benefits of cotton^35,58^ and sugarcane^59^, which are major crops grown in the study area, which could be grown with the amount of water used by *Prosopis*.

### Data reduction and statistical analyses

LoggerNet 4.1 (Campbell Scientific, Inc, Logan UT, USA) was used for downloading sap flow data from data loggers to the laptop and for converting the data to 30 min interval values. EddyPro 6.0 (Licor Nebraska, Lincoln, USA) was employed for processing the high frequency EC data used for calculating ET. Sap flow rates were calculated following Burgess et al^31^. The FAO Penman–Monteith Eq. (^3^) was used to calculate the hourly and daily reference evapotranspiration (ETo) using the weather data. Multiple linear regressions were carried out using either sap flow or ET as response variable and solar radiation, soil moisture, wind speed, vapor pressure deficit and potential ET as explanatory variables at a time in an open source R software version 3.3.3^60^. Maps were made using an open source Quantum GIS (QGIS3.8.3) software^61^.

## Results

### Climatic conditions and diurnal variation in sap flow

**Figure 2:**
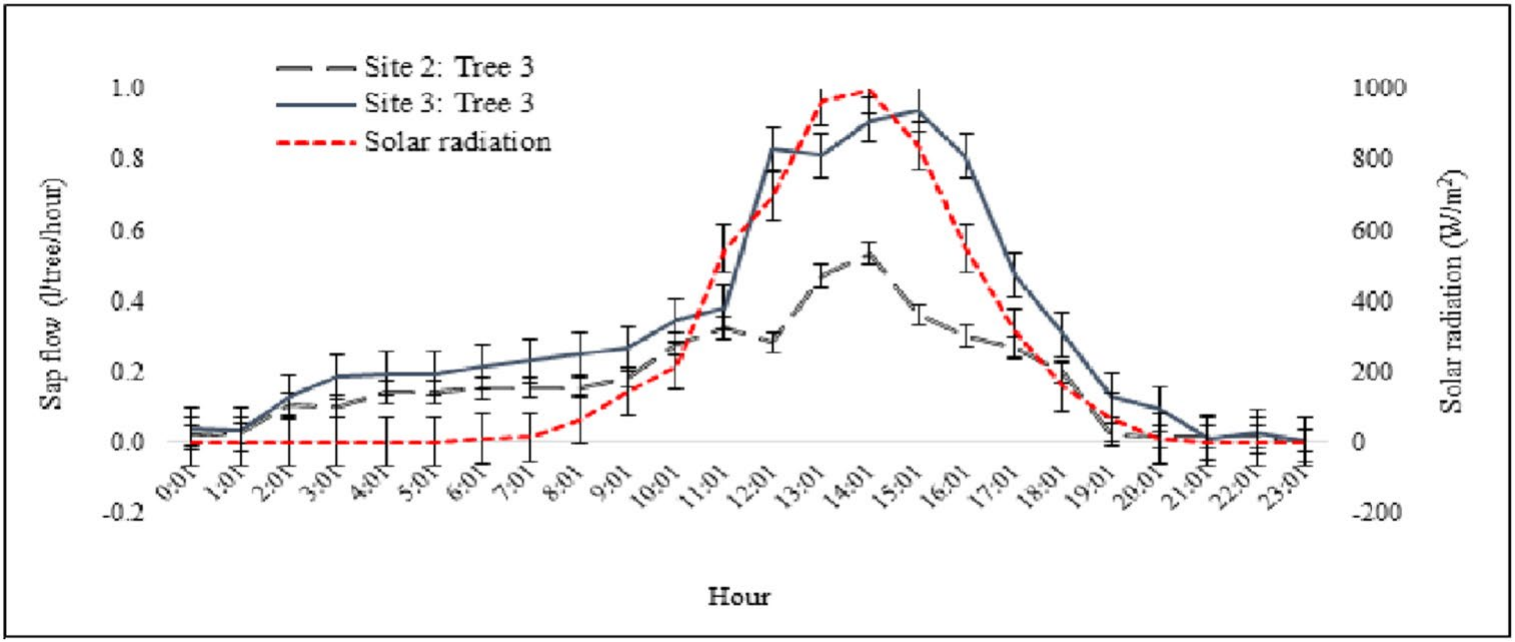
Diurnal sap flow of single *Prosopis* trees in the floodplains (tree measured at site 2) and in the dryland (tree measured at site 3) and daily variation of solar radiation (1st August 2017).

Sap flow varied with the daily solar radiation (Fig. 2) and was the highest at high air temperature and low relative humidity (Appendix 1). The highest solar radiation was registered between 10:00 and 16:00, reaching up to 1000 W/m^2^ around noon, and the daily average varied between 8.1 and 26.5 MJ/m^2^/day. The highest relative humidity was observed from 03:00 to 07:00 while air pressure was more or less constant throughout the day and was about 91 kPa (Appendix 1). When comparing sap flows of trees in floodplains and drylands, the sap flow was lower in the floodplains than in the drylands though the diurnal trends showed similar patterns (Fig. 2).

### Sap flow of Prosopis invasions

#### Stand characteristics and allometric relations

**Figure 3:**
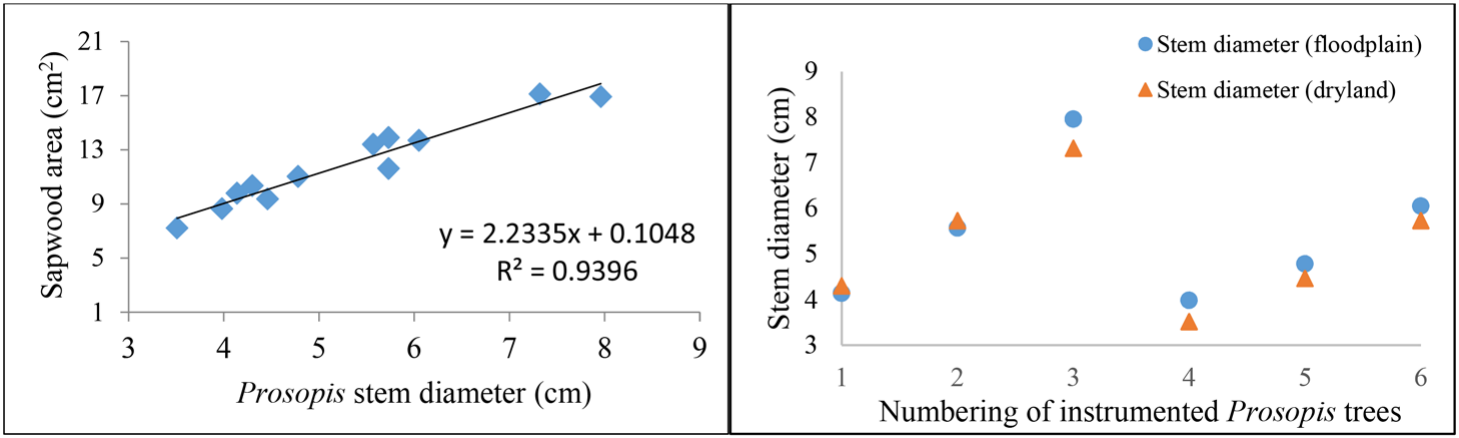
Allometric relationship between sapwood area and stem diameter of the sampled *Prosopis* trees (left) and stem diameter distributions of instrumented trees (right). [Note: tree numbers 1–3 were from the first batch experimental sites, and trees 4–6 were replications of experimental sites for both habitat types, i.e., floodplains and dryland sites].

Sap wood area were found proportional with stem diameters of *Prosopis* tree with R^2^ of 0.94 (Fig. 3, left). Moreover, stem diameters of the instrumented trees were similar between the floodplains and the dryland sites. There was a highly significant relationship between sapwood area and stem diameter of the sampled *Prosopis* trees. The stem diameter distributions were assumed to be representative for trees in the invaded areas (both floodplain and dryland; Fig. 3, right).

#### Sap flow of Prosopis trees

**Figure 4:**
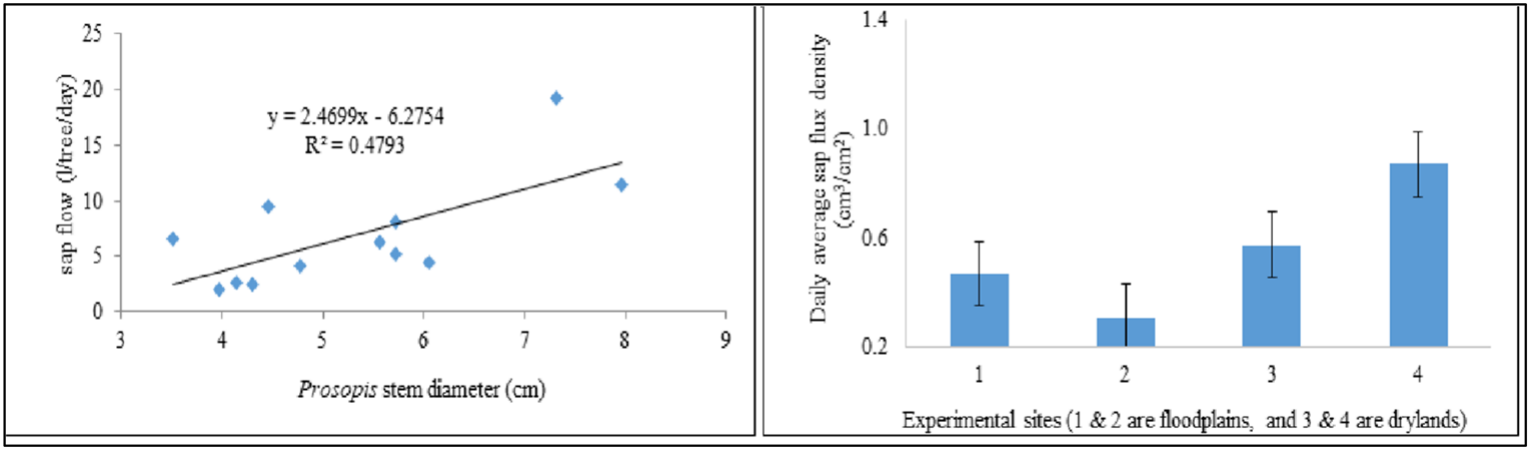
Relationship between sap flow and stem diameter of the sampled *Prosopis* trees (left) and sap flux density per habitat types - floodplains and drylands (right).

**Figure 5:**
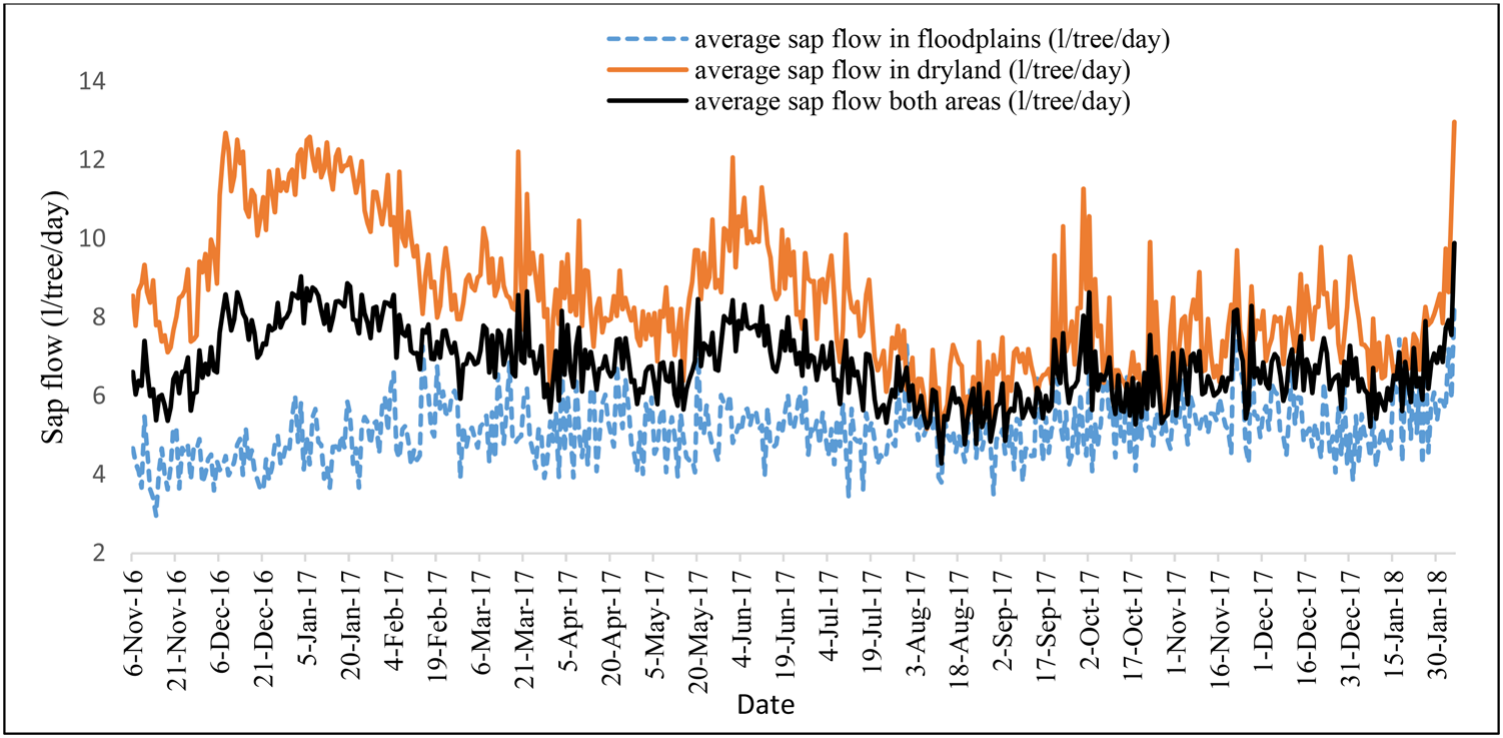
Daily average sap flow of individual *Prosopis* trees across different habitat types (from 6 Nov 2016 to 30 January 2018).

Water use of *Prosopis* trees varied with stem diameter, habitat type (dryland and floodplains), and the seasonal changes in the climatic conditions of the study area. On average, the sampled *Prosopis* trees used about 6.8 ± 2.9 l/tree/day. The instrumented *Prosopis* trees in the floodplains consumed on average less water (5.1 ± 3.4 l/tree/day) than those in the drylands (8.5 ± 5.8 l/tree/day) (Table 1). On the other hand, the relationship between stem diameter and sap flow indicated that stem diameter explained about 48% of the variation in sap flow (Fig. 4, left). After normalizing the daily sap flow by sapwood area, the sap flux density also indicated the same trend that *Prosopis* trees in the dryland area exhibit higher sap flux density than those in the floodplains (Fig. 4, right). Daily average sap flux density (SFD) explained about 64% of the water use of *Prosopis* across the different habitat types. In both cases, we found that trees in the drylands consume more water than those in the floodplains (Fig. 5). Moreover, regression analysis of sap flow as dependent and tree diameter and habitat type as explanatory variables also confirmed this with R^2^ of 0.48, F-stat of 9.22 and significant level of 0.013, which is less than the common *p *value of 0.05 at 95% confidence level. This result, indicates that the water use of *Prosopis* trees in dryland differs significantly from that in the floodplains.

**Table 1:** Daily transpiration dynamics of the instrumented *Prosopis* trees in the floodplains and in the dryland of the Awash River Basin for the period January to November 2017.

Habitat type	Floodplains						Dryland					
Sites	Site 1			Site 2			Site 3			Site 4		
Trees	Tree1	Tree2	Tree3	Tree1	Tree2	Tree3	Tree1	Tree2	Tree3	Tree1	Tree2	Tree3
Max L/tree/day	5.2	17.1	27.8	4.2	11.4	9.7	8.6	16.7	36.8	18.4	22.6	33.2
Min L/tree/day	1.3	2.9	3.1	1.2	1.8	2.2	0.9	2.5	4.1	0.9	3.7	1.8
Avg L/tree/day	2.6	6.2	11.4	1.9	4.17	4.5	2.4	5.1	19.2	6.5	9.5	8.1
(± SD)	(0.7)	(2.1)	(3.9)	(0.4)	(1.3)	(0.9)	(1.1)	(2.5)	(6.4)	(3.9)	(3.2)	(5.7)
Average L by habitat type (± SD)	5.1 (± 3.4) mm/day						8.5 (± 5.8) mm/day					
Average across habitat types (± SD)	6.8 (± 2.9) l/tree/day ~ 3.4 (± 0.5) mm/day*											
ET/day (± SD):	3.7 (± 1.6) mm/day											

*Canopy cover area of *Prosopis* trees was estimated to be on average 2 m^2^.

When comparing seasonal variability between the dry season (January-March) and the rainy season (July–September), *Prosopis* trees were found to use more water during the dry season (7.5 l/tree/day) than during the rainy season (6.0 l/tree/day; Table 2). During the rainy season, the trees sampled in the floodplains used about 5.0 l/tree/day whereas the trees in the drylands used about 7.1 l/tree/day. On the other hand, during the dry season trees in the floodplains and in drylands used about 5.1 and 10.0 l/tree/day, respectively. Similarly, average sap flow across invaded areas reached 3.0 and 3.8 mm per day during the rainy and dry seasons, respectively (Table 2).

**Table 2:** Water use of *Prosopis* trees in floodplains and drylands during the dry and the rainy seasons.

Simple satatistics l/tree/day		Floodplain						Dryland								
		Site 1			Site 2			Site 3			Site 4			Average sap flow		
		Tree1	Tree2	Tree3	Tree1	Tree2	Tree3	Tree1	Tree2	Tree3	Tree1	Tree2	Tree3	Flood plain	Dryland	Both areas
Rainy season (Jul–Sep)	Maximum	4.8	11.5	21.9	3.4	7.5	7.2	8.6	15.2	36.8	14.4	17.4	8.6	7.3	11.3	
	Minimum	1.4	3	5.1	1.3	2.7	2.8	1.6	3.5	4.1	0.9	6.1	1.8	3.4	4.8	
	Average	2.8	6.1	11	1.7	3.8	4.6	3.2	6.3	12.3	4.4	11.5	4.8	5.0	7.1	6.0
	(± SD)	(0.7)	(1.9)	(3.2)	(0.3)	(0.9)	(0.7)	(1.3)	(2.1)	(5)	(3.5)	(2.7)	(1.4)	(0.7)	(1.2)	(0.9)
Dry season (Janl–Mar)	Maximum	4.3	15.8	25.7	2.4	11.4	5.1	2.3	4	33	14.9	10.2	33.2	7.3	12.6	
	Minimum	1.7	2.9	4.3	1.3	2.3	2.9	1.2	2.5	16.6	5.1	4.2	2.4	3.7	5.9	
	Average	2.7	7.0	10.6	1.8	4.5	4.3	1.5	3.2	25.6	8.6	6.5	14.6	5.1	10.0	7.5
	(± SD)	(0.6)	(2.4)	(3.7)	(0.2)	(1.7)	(0.4)	(0.3)	(0.3)	(4.5)	(2.3)	(1.3)	(4.2)	(0.7)	(1.5)	(1.1)

On the other hand, each of the *Prosopis* trees in the floodplains used less water than the trees in the dryland (Appendix 2). Sap flow was plotted across both habitat types and average sap flow (Fig. 5), indicates that dryland has shown higher sap flow for *Prosopis* than floodplains of the same species.

### Evapotranspiration from Prosopis stand

**Figure 6:**
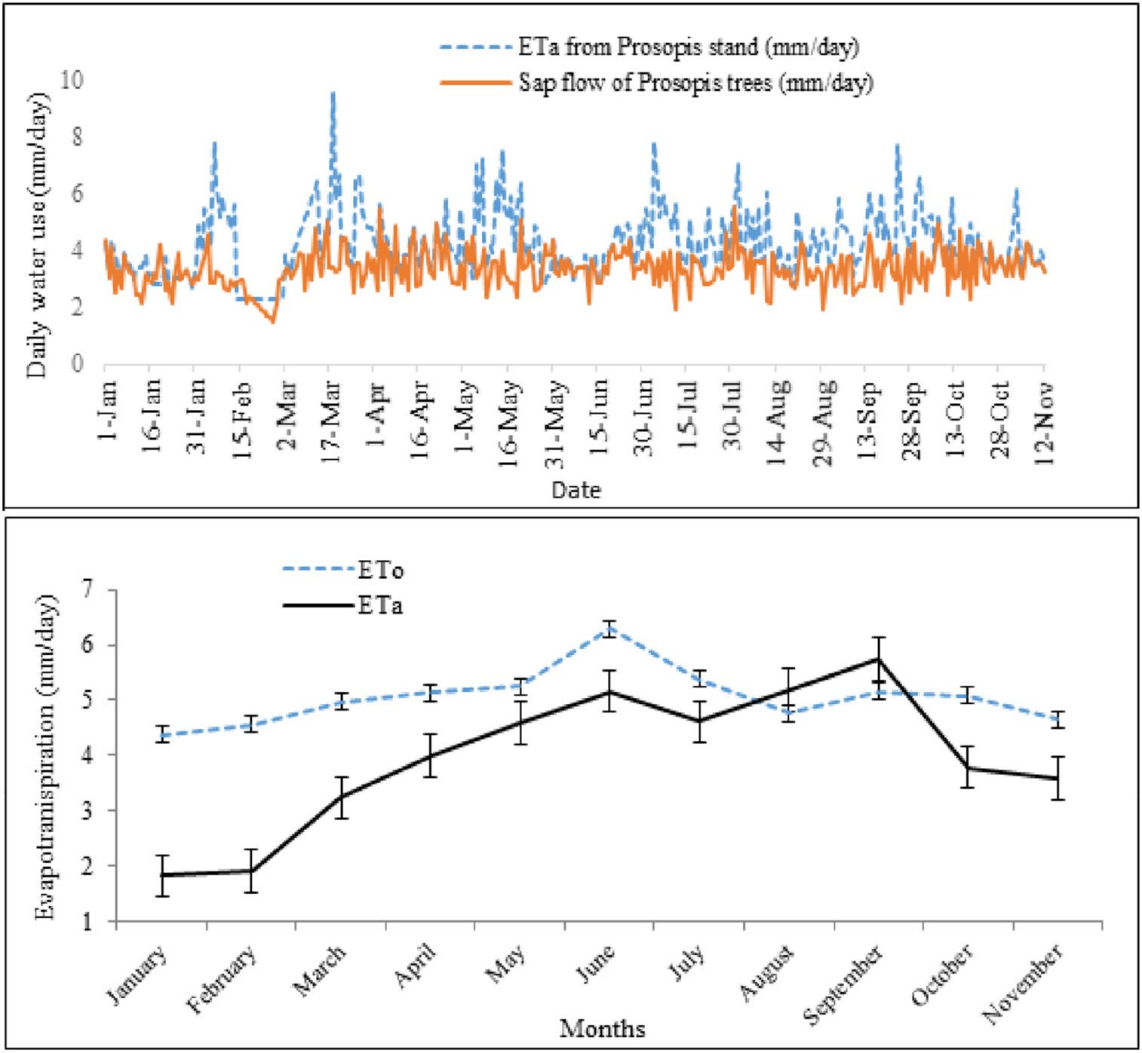
Daily variation in sap flow of individual *Prosopis* trees and actual evapotranspiration ETa of *Prosopis *stands (top), and monthly variation in ETa and potential evapotranspiration (ETo; bottom) from January to November 2017 at experimental site 1.

The daily average of ET_a_ ranged from 1.5 to 9.5 mm, with an annual average of 3.7 mm/day (Fig. 6, top). Moreover, the sap flow and ETa measured at site 1 revealed a similar pattern during the course of the year, although the daily values for ETa were almost always higher than those for sap flow. The average ETo for the 11 months was estimated at 4.99 mm/day. The lowest amount of ET was recorded in January and February due to the low atmospheric evaporative demand while the highest was in June, August and September (Fig. 6, bottom) during summer. Comparing *Prosopis* ETa with the potential evapotranspiration (ETo), the annual average crop coefficient (K_c_) of *Prosopis* was found about 0.77 for *Prosopis* invaded areas.

### Drivers of water use

Regression analyses of possible drivers of water use of *Prosopis* revealed that ETa was explained by wind speed, soil moisture, VPD, and ETo (all p < 0.0001; Table 3A). Sap flow at individual tree level was significantly explained by soil moisture and, VPD and to a lesser extent also by solar radiation and wind speed (Table 3B). Analyses separated by habitat indicated that soil moisture, wind speed, VPD and solar radiation explained a significant amount of variation in sap flow at tree level in drylands, while only VPD and wind speed explained a significant amount of variation in sap flow of *Prosopis* in floodplains (Table 3C & 3D). When combining results from floodplains and drylands, sap flow dynamics at tree level was significantly affected by solar radiation, wind speed, soil moisture and VPD (Table 3E). Crop coefficient of *Prosopis* was also estimated at 0.77 (Table 3A), which is the slope of ETa plotted against ETo.

**Table 3:** Results of multiple linear regression of daily water use of *Prosopis* across 11 months and pedo-climatic factors.

Drivers	Estimate	Std. Error	v t-value	Pr( >|t|)
**(A) Actual evapotranspiration—ETa (mm/day)**^**a**^				
(Intercept)	10.641	1.98635	5.357	< 0.0001***
Solar Radiation	0.029	0.025	1.171	0.242
Wind Speed	0.435	0.091	4.795	< 0.0001***
Soil Moisture	0.091	0.016	5.790	< 0.0001***
VPD	− 1.415	0.223	− 6.334	< 0.0001***
ETo	0.773	0.172	4.508	< 0.0001***
**(B) Sap flow (l/tree/day)**^**b**^				
(Intercept)	1.524	1.253	1.214	0.225
Solar Radiation	0.0418	0.016	2.587	0.010*
Wind Speed	− 0.178	0.057	− 3.109	0.002**
Soil Moisture	− 0.031	0.009	− 3.161	< 0.001***
VPD	0.627	0.141	4.450	< 0.0001***
ETo	0.006	0.108	0.058	0.954
**(C) Sap flow (mm/day) in floodplains**^**c**^				
(Intercept)	0.655	0.610	1.073	0.284
Solar Radiation	0.002	0.007	0.352	0.725
Wind Speed	− 0.056	0.027	− 2.018	0.044*
Soil Moisture	− 0.001	0.004	− 0.250	0.803
VPD	0.199	0.068	2.910	0.003**
ETo	− 0.075	0.052	− 1.435	0.152
**(D) Sap flow (mm/day) in drylands**^**d**^				
(Intercept)	0.418	0.566	0.739	0.460
Solar Radiation	0.019	0.007	2.638	0.008**
Wind Speed	− 0.061	0.025	− 2.385	0.017*
Soil Moisture	− 0.015	0.004	− 3.419	< 0.0001***
VPD	0.214	0.063	3.357	< 0.0001***
ETo	0.045	0.048	0.931	0.352
**(E) Sap flow (mm/day) in both floodplains and drylands**^**e**^				
Intercept)	0.752	0.624	1.206	0.228
Solar Radiation	0.020	0.008	2.582	0.010*
Wind Speed	− 0.091	0.028	− 3.169	< 0.001**
Soil Moisture	− 0.016	0.004	− 3.229	< 0.001**
VPD	0.313	0.0701	4.456	< 0.0001***
ETo	0.008	0.0534	0.150	0.881

^a^Residual standard error: 1.148 on 310 degrees of freedom, Multiple R-squared: 0.3179, Adjusted R-squared: 0.3069, F-statistic: 28.9 on 5 and 310 DF, *p *value: < 2.2e−16.

^b^Residual standard error: 0.7245 on 310 degrees of freedom, Multiple R-squared: 0.1929, Adjusted R-squared: 0.1799, F-statistic: 14.82 on 5 and 310 DF, *p *value: 4.879e−13.

^c^Residual standard error: 0.3531 on 310 degrees of freedom, Multiple R-squared: 0.05818, Adjusted R-squared: 0.04299, F-statistic: 3.83 on 5 and 310 DF, *p *value: 0.002212.

^d^Residual standard error: 0.3276 on 310 degrees of freedom, Multiple R-squared: 0.1547, Adjusted R-squared: 0.1411, F-statistic: 11.35 on 5 and 310 DF, *p *value: 4.59e−10.

^e^Residual standard error: 0.3609 on 310 degrees of freedom, Multiple R-squared: 0.1946, Adjusted R-squared: 0.1816, F-statistic: 14.98 on 5 and 310 DF, *p *value: 3.521e−13; Asterisks indicate level of significance: *** =  < 0.001, ** < 0.01, * =  < 0.05.

When comparing climatic factors as drivers of water use and measured water uses (in terms of sap flow and ETa), ETa was found the lowest when wind speed was low while sap flow of trees in the drylands changed with VPD, soil moisture, and with solar radiation, but not with wind speed. It was also observed that the higher the solar radiation, the higher the sap flow (Fig. 2; Appendix 3).

### Upscaling Prosopis water use moderation to the Afar Regional level

**Figure 7:**
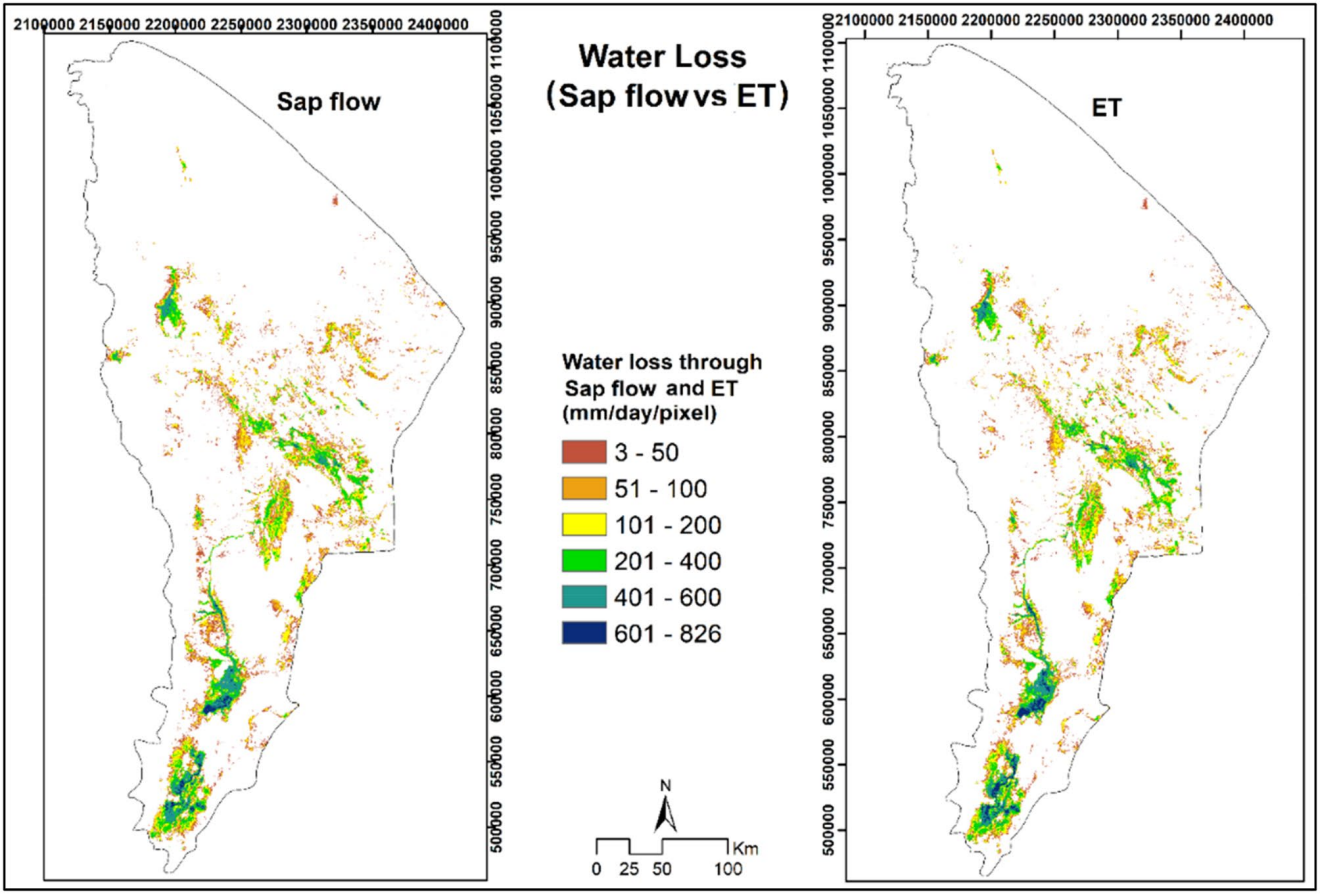
Water loss over areas invaded by *Prosopis* stands through sap flow of individual *Prosopis* trees (left side) and ETa (right side)

Depending on *Prosopis* cover, sap flow and ETa were estimated to range between 3 and 826 mm/day at a pixel size of 15 × 15 m (or the maximum value of 826 mm/day/pixel, is approximately equivalent to 3.67 mm) (Fig. 7). Upscaling the results from sap flow of *Prosopis* trees and ETa of *Prosopis* stand from pixels to the regional scale revealed that *Prosopis* consumed about 3.1 and 3.3 billion m^3^ per year, respectively, over the invaded areas of 1.18 million ha in Afar Region, which approximates to 279 mm per year of water use and it is about 50% of the annual precipitation of the region.

Based on current regional water tariffs, the economic impact of water loss due to *Prosopis* invasion in the Afar Region was estimated at US$ 465,000—500,000 per year. Considering that cotton production requires some 7,000 m^3^/ha/year (consultation with experts), the water consumed by *Prosopis* corresponds to the amount of water needed to grow approximately 460,000 ha of cotton with an average crop yield of 2.1 ton/ha^35^ and an average market price of US$ 500–600/ton (derived from Alebel et al^35^), depending on the market prices for different cotton grades. Thus, the water used by *Prosopis* can produce approximately 674,500 tons of cotton with a market value of US$ 0.95 to 1.0 billion per growing season. With a benefit to cost ratio of 1.49 reported for cotton production in Afar region^58^, this results in a net benefit of approx. US$ 310–330 million per a growing season. Similarly, considering a water use for sugar cane of some 15,000 m^3^/ha/year and an average yield of approx. 37 tons sugar/ha^35^, the water used by *Prosopis* would allow irrigation of some 210,000 ha of sugar cane, which would yield approx. 7,8 million tons of sugar. With a market price of an average of US$ 536/ton sugar and a net benefit to cost ratio of 1.12 was obtained from sugar cane production with optimal fertilizer application^59^, this results in a market value of approximately 4,2 billion US$ and a net benefit of approx. US$ 470 million per growing season.

## Discussion

The results of this study indicate that the invasive evergreen *Prosopis* uses a significant amount of water (3.1 to 3.3 billion m^3^ per year over invaded areas) in the Afar Region. Considering that the annual precipitation in the study region is approximately 560 mm^29^, our findings indicate that *Prosopis* consumes about half of the amount of water received from precipitation in the invaded area in the Afar Region (Appendix 1).

### Sap flow in individual trees and evapotranspiration from stands of Prosopis

Our study revealed that variation in sap flow among *Prosopis* trees in Afar Region was affected by sapwood area, stem diameter, habitat type and by climatic factors. This finding concurs with Dzikiti et al^22^ that reported a close relationship between sapwood areas and stem diameter as well as between stem diameter and sap flow at the tree level from invasive species, leaf-shedding *Prosopis* species in South Africa. Tree hydrology is largely controlled by species traits, the regional climatic characteristics and dynamics of water and energy inputs during the growing season^6^.

The higher solar radiation, vapor pressure deficit and air temperature in the drylands may thus favor higher water use by *Prosopis* trees growing in this habitat, relative to trees growing in the floodplains. The higher sap flow of evergreen *Prosopis* trees in the drylands of Awash River Basin may therefore be explained by the fact that the study area is a flat plain which may provide a sufficiently large groundwater reservoir from which deep-rooting trees of *Prosopis* can take up water also during the dry season, as groundwater can be found within the range of 10–20 m depth and the depth increased as we move away from the river (expert consultation). Le Maitre et al.^9^ reported that in South Africa tree water use of leaf-shedding *Prosopis* species is higher in riparian settings or shallow water tables where water availability is greater, relative to dryland settings. They therefore concluded that the impact of *Prosopis* on ecosystems services is greater in riparian than in dryland ecosystems. Similarly, in the southern parts of the USA water use by *Prosopis velutina* Wooton tended to be higher in the floodplains than in adjacent dryland (Scott et al., 2006). In contrast, the present study revealed that sap flow of the evergreen *Prosopis juliflora* trees was higher in the drylands than in the floodplains of Awash River Basin. This contradicts the general assumption that trees growing in areas with wet soils consume more water than trees growing in dry soil. While rainwater is the preferred source of *Prosopis* when available, Dzikiti et al.^14,22^ showed that in South Africa *Prosopis* switched to tapping groundwater once rainwater sources were exhausted. Fritzsche et al^39^ found that in South Ethiopia growth of the exotic *Eucalyptus globulus* Labill. was largely independent of topsoil water content, because tree has the flexibility to resort to groundwater if necessary.

In general, tree water use is expected to vary between the wet and the dry season. In semi-arid to arid regions such as Afar, water availability during the wet season is high and VPD is relatively low. During the dry season, when soil moisture is low and VPD, temperature and solar radiation are high, tree water use should decline to prevent canopy desiccation. However, in contrast to the studies cited above, in savannas of northern Australia tree water use was found to either not differ between wet and dry seasons^40^, or even increase during the dry season^41^. This result was explained on the basis of the fact that rooting depth allowed the plant access to sufficient water stored in the soil also during the dry season to enable transpiration to proceed. Similarly, the study found that the evergreen *P.juliflora* used more water and transpired more during the dry season than during the rainy season. Tanaka et al^42^ showed that water use of trees in a hill evergreen forest in Thailand peaked in the late dry season. The factors contributing to higher water use of trees in drylands relative to the floodplains may thus also explain why *Prosopis* in Afar consumes more water during the dry than during the wet season. Hence, the findings suggest that the evergreen leaf feature of *P. juliflora* not only leads to water abstraction from the groundwater during both the wet and the dry season, but also it even increases water abstraction during the dry season, which significantly exacerbates regional water shortage.

Total ET is acquired from three different sources, namely soil evaporation, interception loss and transpiration, which are mainly influenced by the vegetation composition. For example, the degree of ground cover has a substantial influence on soil evaporation^43^. In this study, however, soil evaporation contributed little to total ET, which might be due to the fact that the soil is very dry and understorey transpiration is low because of absence of undergrowth in the study area. However, the degree of soil evaporation, in turn, is determined by the meteorological conditions^44,45^. In this study ET varied with microclimate, particularly precipitation and its effect on soil moisture and wind speed, whereas sap flow varied with habitat type, solar radiation and sap wood area of *Prosopis* trees. These results thus suggest that management attempts to reduce water loss due to *Prosopis* invasion should primarily target invasive trees in the drylands where trees consume particularly high amounts of water, also during the dry season.

The water use of invasive species is of concern in species conservation or ecosystem restoration programs in semi-arid and arid ecosystems, as well as when adapting to climate change by reducing competition for the often scarce water resources^9,14^. *Prosopis* invasions are having a negative effect on the stability of native tree populations in South Africa and are linked to increased mortality of native trees^46^. While *Prosopis* has been reported to have allelopathic effects^47^, it is likely that dense thickets of *Prosopis* also impact native vegetation through competition for water^48^.

Various studies indicate that, although individual native and invasive tree species may have similar sap flow rates, but that invasive-dominated ecosystems were more likely to have higher sap flow rates per unit area than native-dominated ecosystems because invasive species grow in denser stands than natives and dense stands consume more water than sparsely stocked stands^13,14,36^. Dzikiti et al^14^ reported that in South Africa *Prosopis* stands transpired five times more water (544 mm/y) than *Vachellia karroo* (Hayne) Banfi & Galasso stands (91 mm/y). The results of the present study suggest that in the tropical Afar Region *Prosopis* stands with 100% cover transpired about 1241—1350 mm/y, which is about six times more water than used by *Prosopis* stands in South Africa^12^. The results of Dzikiti et al^14^ and this study were based on a shared methodology and should therefore be comparable. Nevertheless, there may be several other factors affecting the significantly higher transpiration revealed by the current study. First, the invasive species in Ethiopia and other parts of the tropical region in Eastern Africa, *P. juliflora*, is the evergreen plant which uses water throughout the year for its photosynthesis activities, whereas the *Prosopis* taxa in South Africa shed leaves during the cold seasons and are thus likely to have periods with low transpiration and evapotranspiration. Secondly, *Prosopis* in Afar builds very dense thickets, due to past and partly ongoing utilization for charcoal production, which created multi-stemmed trees that use more water. Also, the deep-rooted *Prosopis* may benefit from the alluvial nature of the soil in Afar region which allows easy penetrations of the soil layers so as to abstract more water than native species do, although sandy soils drain more water to the groundwater table. This type of soil, in turn, forces the tree to grow deep tap-root systems in search for more water.

Finally, comparing three scenarios for crop specific coefficients (grass reference at standard height of 0.5 m, bare soil and *Prosopis* invaded areas scenarios), bare soil have a crop coefficient (Kc) of 0.3 to 0.5 while well-watered grass has 0.7 to 1.0^3^ whereas *Prosopis* invaded areas exhibited a Kc of about 0.77 all year round, indicating that invaded areas extracted more groundwater on an annual basis as grass-dominated habitats in the study area consume water only during the 2–3 months of the rainy season and would dry up during dry seasons, unlike the areas invaded by evergreen *Prosopis* in Ethiopia.

### Upscaling Prosopis water use moderation to the Afar Regional level

Understanding the effect of vegetation change on transpiration and ET across habitat types (in our case drylands and floodplains) is important for scaling up since ET plays an important role in the water balance of hydrological catchments or landscapes^49,50^. Similar to our approach, previous studies assessing forest canopy transpiration or evapotranspiration at the water catchment level used average species sap flux values and/or ET estimates per unit ground area across habitat types to scale-up to the landscape level^51^). Fractional cover maps, as the one used in this study^26^ reflect not only the current distribution but also the different levels of invasion across the region and are thus more accurate in upscaling impact at the water catchment or ecosystem level than presence/absence maps^52^. The *Prosopis* map we used in the present study for upscaling water consumption by *Prosopis* was developed using different driving factors (environmental, climatic, remote sensing and other important factors for its distribution) implemented on machine learning algorithms as species spatial patterns are important for understanding spatial estimates of transpiration. This is consistent with Loranty et al^51^ that incorporated atmospheric and edaphic drivers to upscale sap flow from trees and ET from stands level to an aspen-dominated forest across a upland-to-wetland gradient, and doing so in a spatial context, that achieved a more accurate estimate.

The findings of this study indicate that between 8.3–9.1 billion liters of water per day are consumed by *Prosopis* in the invaded areas of the arid and semi-arid Afar Region. Hence, this fast-growing exotic tree is likely to exacerbate the effects of climate change on the provisioning of ecosystem services through its impact on ecosystem water budget. Based on the extremely low current water tariff for water by the Awash Basin Authority, which can be considered as a payment for ecosystems service by investors, the economic value of water used by *Prosopis* potentially amounts to US$ 465,000 – 500,000 per year across the invaded range, which is equivalent to approximately 15—16 million Eth Birr per year. When assessing the net benefits of water use by *Prosopis* in Afar in terms of market prices of cotton and sugar cane production capacity, the value of the water consumed by *Prosopis* is much higher than that estimated from the water tariffs. As noted by Le Maitre et al^36^, there is a lack of studies assessing the financial impacts of IAPS on yields, which may lead to an underestimation of the economic benefits of managing IAPS for society^53^.

Up-scaling of the water use of *Prosopis* to the regional scale has immense implications to decision makers and land use planners. The Awash River is known to be a losing river system as the water abstracted by vegetation, mainly crop plants and IAPS, can be regarded as an abstraction from the river recharging system to the groundwater resource. Extensive abstraction of groundwater has serious consequences on the water availability to downstream, as well. Moreover, this study revealed that *Prosopis* uses the groundwater throughout the year as the annual rainfall is by far less than the water consumed by *Prosopis*, which affects water availability to other services by influencing ecosystem water budget that could be used for the provision of other ecosystem services and thus for supporting rural livelihoods, including crop production or fodder for livestock. Dzikiti et al^22^ showed that the decline in groundwater level under *Prosopis* stands can be attributed to the active water uptake by the trees to meet their transpirational demand. In addition, dense thickets of thorny *Prosopis* trees create physical barriers for domestic and wild animals to access surface water^19,54^. Moreover, *Prosopis* affects water availability for indigenous trees and herbaceous species, which also contributes to a degradation of ecosystem functioning and the provision of ecosystem services^55^. For example, indigenous palatable grasses species such as *Chrysopogon plumulosus* (Durfu)*, Cenchrus ciliaris* (Serdoitas) *and Seataria acromelaena* (Mussa) and multipurpose trees species such as *Acacia tortilis* (Eebto), *A. senegal* (Adebo), *A. nilotica* (Keselto) *and Dobera gelabera* (Gersaito) are threatened by *Prosopis* invasion^18,56^. Thus, there is a need for sustainable management of *Prosopis* invasion in order to secure benefits from groundwater resources and other services provided by the ecosystems in this semi-arid and arid region.

We acknowledge that the up-scaling of the water use of *Prosopis* to the regional scale is based on a limited number of experimental sites and some assumptions that entail a certain level of uncertainty. All the four experimental sites were situated in the southern part of Afar Region and may therefore not be representative of the total area invaded by *Prosopis* (Fig. 1). However, climatic conditions are similar across the lowlands of Afar Region, i.e. the area invaded by *Prosopis*, with the same rainy seasons and comparable average temperatures across the year. Another caveat of our study is that the upscaling of ETa was based on measurements taken at one experimental site only. As it was not feasible to measure ETa at all four experimental sites, we triangulated it with sap flow values at site 1 and found that our ETa values were reasonable. Nevertheless, investigations on the variation in water use of *Prosopis* across the invaded range in Afar Region as well as in neighboring countries would further improve our understanding of the impact of *Prosopis* on the water balance of semi-arid and arid ecosystems in the tropical regions of Eastern Africa.

## Conclusions

While *Prosopis* trees were, among others, introduced in Eastern Africa to increase the availability of wood and to stabilize soil in degraded ecosystems, their invasion has been identified as a serious challenge to biodiversity, environment and the rural livelihoods^15,18,20,30,54,55^. In the context of climate change and an increasing frequency of drought events in dry regions of Sub-Saharan Africa, there is increasing evidence that invasive trees can increase transpiration and ET losses and thus reduce surface water or groundwater recharge^36^. The results of this study provide evidence that in the Afar Region the evergreen *P. juliflora* not only consumes water throughout the year, but even consumes more water during the dry than during the rainy season. Thus, unless the spread of *P. juliflora* is contained and the density reduced in areas where it has become established, this invasive tree is likely to have serious consequences for sustainable livelihoods in the region. The estimated net benefit alone would strongly justify the implementation of a coordinated control program.

## Supplementary information

Supplementary information

## References

[1] Vörösmarty CJ, Green P, Salisbury J, Lammers RB (2000). Global water resources: Vulnerability from climate change and population growth. Science.

[2] Mekonnen MM, Hoekstra AY (2016). Sustainability: four billion people facing severe water scarcity. Sci. Adv..

[3] Allen RG (1998). FAO irrigation and drainage paper crop by. Irrig. Drain..

[4] Allen RG, Tasumi M, Morse A, Trezza R (2005). A landsat-based energy balance and evapotranspiration model in Western US water rights regulation and planning. Irrig. Drain. Syst..

[5] Gebler S (2015). Actual evapotranspiration and precipitation measured by lysimeters: a comparison with eddy covariance and tipping bucket. Hydrol. Earth Syst. Sci..

[6] Sun G, Noormets A, Chen J, McNulty SG (2008). Evapotranspiration estimates from eddy covariance towers and hydrologic modeling in managed forests in Northern Wisconsin USA. Agric. For. Meteorol..

[7] Farley KA, Jobbágy EG, Jackson RB (2005). Effects of afforestation on water yield: A global synthesis with implications for policy. Glob. Chang. Biol..

[8] Jackson RB (2005). Trading water for carbon with biological carbon sequestration. Science.

[9] Le Maitre, D. C., Gush, M. B. & Dzikiti, S. Impacts of invading alien plant species on water flows at stand and catchment scales. *AoB Plants* **7**, (2015). 10.1093/aobpla/plv043 PMC4480063 25935861

[10] Masters, G., Norgrove, L. Climate change and invasive alien species. *CABI Working Paper*. **1**, 1–31 (2010).

[11] Vilà M (2011). Ecological impacts of invasive alien plants: a meta-analysis of their effects on species, communities and ecosystems. Ecol. Lett..

[12] Robinson TP, van Klinken RD, Metternicht G (2008). Spatial and temporal rates and patterns of mesquite (Prosopis species) invasion in Western Australia. J. Arid Environ..

[13] Cavaleri MA, Ostertag R, Cordell S, Sack L (2014). Native trees show conservative water use relative to invasive trees : results from a removal experiment in a Hawaiian wet forest. Conserv. Physiol..

[14] Dzikiti S (2017). Forest ecology and management assessing water use by prosopis invasions and vachellia karroo trees: implications for groundwater recovery following alien plant removal in an arid catchment in South Africa. For. Ecol. Manage..

[15] Shackleton RT, Le Maitre DC, Pasiecznik NM, Richardson DM (2014). Prosopis: a global assessment of the biogeography, benefits, impacts and management of one of the world’s worst woody invasive plant taxa. AoB Plants.

[16] Pasiecznik, N.M., Felker, P., Harris, P.J.C., Harsh, L.N., Cruz, G., Tewari, J.C., Cadoret, K. and Maldonado, L.J. The Prosopis juliflora - Prosopis pallida Complex: A Monograph. *HDRA*, 1–172 (2001).

[17] Boy, G. & Witt, A. Invasive Alien Plants and Their Management in Africa. UNEP/GEF Removing Barriers to Invasive Plant Manag. *CABI Africa, International Coordination Unit.*, 1–184(2013). https://www.cabi.org/.

[18] Engda, G. Spatial and temporal analysis of *Prosopis juliflora* ( Swarz ) DC invasion in Amibara woreda of the Afar NRS spatial and temporal analysis of Prosopis juliflora ( Swarz ) DC Invasion in Amibara Woreda of the Afar NRS. MSc Thesis, Addis Ababa University, (2009).

[19] Wakie TT, Hoag D, Evangelista PH, Luizza M, Laituri M (2016). Is control through utilization a cost effective Prosopis juliflora management strategy?. J. Environ. Manag..

[20] Edmund, T. et al. Direct and indirect effects of invasive species: biodiversity loss is a major mechanism by which an invasive tree affects ecosystem functioning. 1–13 (2019). doi:10.1111/1365-2745.13268

[21] Dzikiti S, Ntshidi Z, Le Maitre DC, Bugan RDH, Mazvimavi D, Schachtschneider K, Jovanovic NZ, Pienaar HH (2017). Assessing water use by Prosopis invasions and Vachellia karroo trees: Implications for groundwater recovery following alien plant removal in an arid catchment in South Africa. For. Ecol. Manage..

[22] Dzikiti S, Schachtschneider K, Naiken V, Gush M, Moses G, Le Maitre DC (2013). Water relations and the effects of clearing invasive Prosopis trees on groundwater in an arid environment in the Northern Cape South Africa. J. Arid Environ..

[23] van Wilgen BW, Wannenburgh A (2016). Co-facilitating invasive species control, water conservation and poverty relief: achievements and challenges in South Africa’s Working for Water programme. Curr. Opin. Environ. Sustain..

[24] Mcshane RR (2015). Distribution of invasive and native riparian woody plants across the western USA in relation to climate, river flow, floodplain geometry and patterns of introduction. Ecography (Cop.).

[25] Webb RH, Leake SA (2006). Ground-water surface-water interactions and long-term change in riverine riparian vegetation in the southwestern United States. J. Hydrol..

[26] Shiferaw H, Schaffner U, Bewket W, Alamirew T, Zeleke G, Teketay D, Eckert S (2019). Modeling Prosopis invasion and impacts on ecosystem services and rural livelihoods in the Afar Region. Sci. Rep..

[27] Kidanewold BB, Seleshi Y, Melesse AM (2014). Surface water and groundwater resources of Ethiopia: potentials and challenges of water resources development chapter6 surface water and groundwater resources of Ethiopia: potentials and challenges of water resources development. Chapter.

[28] Authority, A. B. Executive summary of strategic river basin plan for Awash basin. (2017).

[29] Shiferaw H, Teketay D, Nemomissa S, Assefa F (2004). Some biological characteristics that foster the invasion of Prosopis juliflora (Sw.) DC. at Middle Awash Rift Valley Area, north-eastern Ethiopia. J. Arid Environ..

[30] Shiferaw H, Bewket W, Alamirew T, Zeleke G, Teketay D, Bekele K, Schaffner U, Eckert S (2019). Implications of land use/land cover dynamics and Prosopis invasion on ecosystem service values in Afar Region Ethiopia. Sci. Total Environ..

[31] Burgess SS (2001). An improved heat pulse method to measure low and reverse rates of sap flow in woody plants. Tree Physiol..

[32] Scott RL, Cable WL, Hultine KR (2008). The ecohydrologic significance of hydraulic redistribution in a semiarid savanna. Water Resour. Res..

[33] Shiferaw H, Bewket W, Eckert S (2019). Performances of machine learning algorithms for mapping fractional cover of an invasive plant species in a Dryland ecosystem. Ecol. Evol..

[34] Ayana M, Teklay G, Abate M, Eshetu F (2015). Irrigation water pricing in Awash River Basin of Ethiopia : evaluation of its impact on scheme-level irrigation performances and willingness to pay. Afr. J. Agric. Res..

[35] Weldesilassie AB, Bekele F, Gebrehiwot BA (2014). An institutional assessment of the cotton and sugarcane commodities in Ethiopia: the climate change perspective. EDRI Res. Rep..

[36] Le Maitre, D., Blignaut, J., Clulow, A., Dzikiti, S., Everson C., Görgens A., and Gush, M. Impacts of plant invasions on terrestrial water flows in South Africa. In: Wilgen, B. W. Van, Measey, J., Richardson, D. M. & Wilson, J. R. *Biological invasions in South Africa,* **431**–458, Ch15 (2020)

[37] Scott RL, Huxman TE, Williams DG, Goodrich DC (2006). Ecohydrological impacts of woody-plant encroachment: Seasonal patterns of water and carbon dioxide exchange within a semiarid riparian environment. Glob. Chang. Biol..

[38] Dzikiti S (2013). Water relations and the effects of clearing invasive Prosopis trees on groundwater in an arid environment in the Northern Cape South Africa. J. Arid Environ..

[39] Fritzsche F (2006). Soil-plant hydrology of indigenous and exotic trees in an Ethiopian montane forest. Tree Physiol..

[40] Eamus D, Grady APO, Hutley L (2007). Dry season conditions determine wet season water use in the wet – dry tropical savannas of northern Australia. Tree Physiol..

[41] Grady APO, Eamus D, Hutley LB (1999). Transpiration increases during the dry season: patterns of tree water use in eucalypt open-forests of northern Australia. Tree Physiol..

[42] Tanaka K, Takizawa H, Tanaka N, Kosaka I, Yoshifuji N, Tantasirin C, Piman S, Suzuki M, Tangtham N (2003). Transpiration peak over a hill evergreen forest in northern Thailand in the late dry season: assessing the seasonal changes in evapotranspiration using a multilayer model. J. Geogr. Res..

[43] Merta M, Seidler C, Fjodorowa T (2006). Estimation of evaporation components in agricultural crops. Biologia (Bratisl)..

[44] Gardiol JM, Serio LA, Della Maggiora AI (2003). Modelling evapotranspiration of corn (Zea mays) under different plant densities. J. Hydrol..

[45] Lund MR, Soegaard H (2003). Modelling of evaporation in a sparse millet crop using a two-source model including sensible heat advection within the canopy. J. Hydrol..

[46] Shackleton RT, Le Maitre DC, Van Wilgen BW, Richardson DM (2015). The impact of invasive alien Prosopis species (mesquite) on native plants in different environments in South Africa. South Afr. J. Bot..

[47] El-Keblawy A, Al-Rawai A (2007). Impacts of the invasive exotic Prosopis juliflora (Sw.) D.C. on the native flora and soils of the UAE. Plant Ecol..

[48] Schachtschneider K, February EC (2013). Impact of Prosopis invasion on a keystone tree species in the Kalahari Desert. Plant Ecol..

[49] Zhang L, Dawes WR, Walker GR (2001). Response of mean annual evapotranspiration to vegetation changes at catchment scale. Water Resour. Res..

[50] Hagyó A, Rajkai K, Nagy Z (2006). Effect of forest and grassland vegetation on soil hydrology in Mátra effect of forest and grassland vegetation on soil hydrology in Mátra Mountains ( Hungary). Biologia.

[51] Loranty MM, Mackay DS, Ewers BE, Adelman JD, Kruger EL (2008). Environmental drivers of spatial variation in whole-tree transpiration in an aspen-dominated upland-to-wetland forest gradient. Water Resour. Res..

[52] Wakie TT, Evangelista PH, Jarnevich CS, Laituri M (2014). Mapping current and potential distribution of non-native prosopis juliflorain the Afar region of Ethiopia. PLoS ONE.

[53] Mudavanhu S (2017). A cost-benefit analysis of clearing invasive alien plants in the Berg River quaternary catchment of South Africa. J. Agric. Resour. Econ..

[54] Ayanu Y (2014). Ecosystem engineer unleashed: Prosopis juliflora threatening ecosystem services?. Reg. Environ. Chang..

[55] Keller, R.P., Lodge, M., Lewis, M.A., Shogren J.F. (Eds) Bioeconomics of invasive species: integrating ecology, economics, policy, and management. *Oxford University Press*, Oxford, 1–285 (2009)

[56] Shackleton RT, Le Maitre DC, Van Wilgen BW, Richardson DM (2015). The impact of invasive alien Prosopis species (mesquite) on native plants in different environments in South Africa. South Afr. J. Bot..

[57] Swanson RH, Whitfield DWA (1981). A numerical analysis of heat pulse velocity theory and practice. J. Exp. Bot..

[58] Belay G, Yami M, Bekele D (2020). Analysis of costs of production and profitability for irrigated cotton under smallholder production systems; the case of middle awash valley. Ethiop. J. Agric. Sci..

[59] Teshome Z, Abejehu G, Hagos H (2014). Effect of nitrogen and compost on sugarcane (Saccharum Officinarum L) at Metahara sugarcane plantation. Adv. Crop. Sci. Technol..

[60] R Core Team. R: A language and environment for statistical computing. Vienna, Austria: R Foundation for Statistical Computing, 3.3.3. (2017). Retrieved from http://www.R-project.org

[61] Quantum GIS (QGIS). https://www.qgis.org/en/site/.

