# Supplementary material for: Water use of Prosopis juliflora and its impacts on catchment water budget and rural livelihoods in Afar Region, Ethiopia
Source: Sci Rep. 2021 Jan 29;11:2688. doi: 10.1038/s41598-021-81776-6 (PMC7846740; doi:10.1038/s41598-021-81776-6)

**Water use of *Prosopis juliflora* and its impacts on catchment water budget and rural livelihoods in Afar Region, Ethiopia**

Hailu Shiferaw^1,2#^, Tena Alamirew^2^, Sebinasi Dzikiti^3,5^, Woldeamlak Bewket^1^, Gete Zeleke^2^ and Urs Schaffner^4^

^1^Department of Geography and Environmental Studies, Addis Ababa University, P.O. Box 1176, Ethiopia

^2^Water and Land Resource Centre, Addis Ababa University, P.O. Box 3880, Addis Ababa, Ethiopia

^3^CSIR Smart Places Cluster, 11 Jan Cilliers Street Stellenbosch 7599, South Africa

^4^CABI Rue des Grillons 1, CH-2800 Delémont, Switzerland

^5^ Department of Horticultural Science, University of Stellenbosch, P. Bag X1 Matieland Stellenbosch, 7602, South Africa

**Appendices**

| Appendix 1. Average monthly climatic variables | | | |  |  |
| --- | --- | --- | --- | --- | --- |
| Month | Average RH (%) | Average Temp (°C) | Average Pressure (kPa) | Average Rainfall (mm) | Solar W/m2/month |
| 10/23/2016 | 31.0 | 27.8 | 91.6 | 0.0 | 11794.4 |
| 11/1/2016 | 39.6 | 25.9 | 91.7 |  |  |
| 12/1/2016 | 38.5 | 24.7 | 91.8 |  |  |
| 1/1/2017 | 28.2 | 23.8 | 91.7 | 0.0 | 12333.3 |
| 2/1/2017 | 50.2 | 26.5 | 91.6 | 7.6 | 11707.2 |
| 3/1/2017 | 40.4 | 29.0 | 91.4 | 47.6 | 13302.8 |
| 4/1/2017 | 36.9 | 30.4 | 91.4 | 1.4 | 13126.0 |
| 5/1/2017 | 52.4 | 29.0 | 91.4 | 94.2 | 12251.4 |
| 6/1/2017 | 33.9 | 32.0 | 91.2 | 11.0 | 12203.7 |
| 7/1/2017 | 50.9 | 29.1 | 91.2 | 120.4 | 10990.7 |
| 8/1/2017 | 55.2 | 28.0 | 91.2 | 146.6 | 10592.2 |
| 9/1/2017 | 56.0 | 27.8 | 91.3 | 46.2 | 11407.0 |
| 10/1/2017 | 40.6 | 28.5 | 91.4 | 1.8 | 11668.3 |
| 11/1/2017 | 37.2 | 26.3 | 91.6 | 0.0 | 11693.0 |
| 12/1/2017 | 34.0 | 22.8 | 91.8 |  |  |
| 1/1/2018 | 37.8 | 23.8 | 91.6 |  |  |
| 2/1/2018 | 41.1 | 26.8 | 91.5 |  |  |
| 3/1/2018 | 37.8 | 28.1 | 91.4 |  |  |
| 4/1/2018 | 52.7 | 28.3 | 91.3 |  |  |
| 5/1/2018 | 39.3 | 30.8 | 91.2 |  |  |
| 6/1/2018 | 39.2 | 31.4 | 91.2 |  |  |

Appendix 2. Daily sap flow (l/tree/day) from 12 instrumented *Prosopis* trees: six trees from floodplains (site 1 & site 2, top), and six trees from drylands (site 3 & site 4, bottom).

Appendix 3. Matrix of associations of multiple linear regression


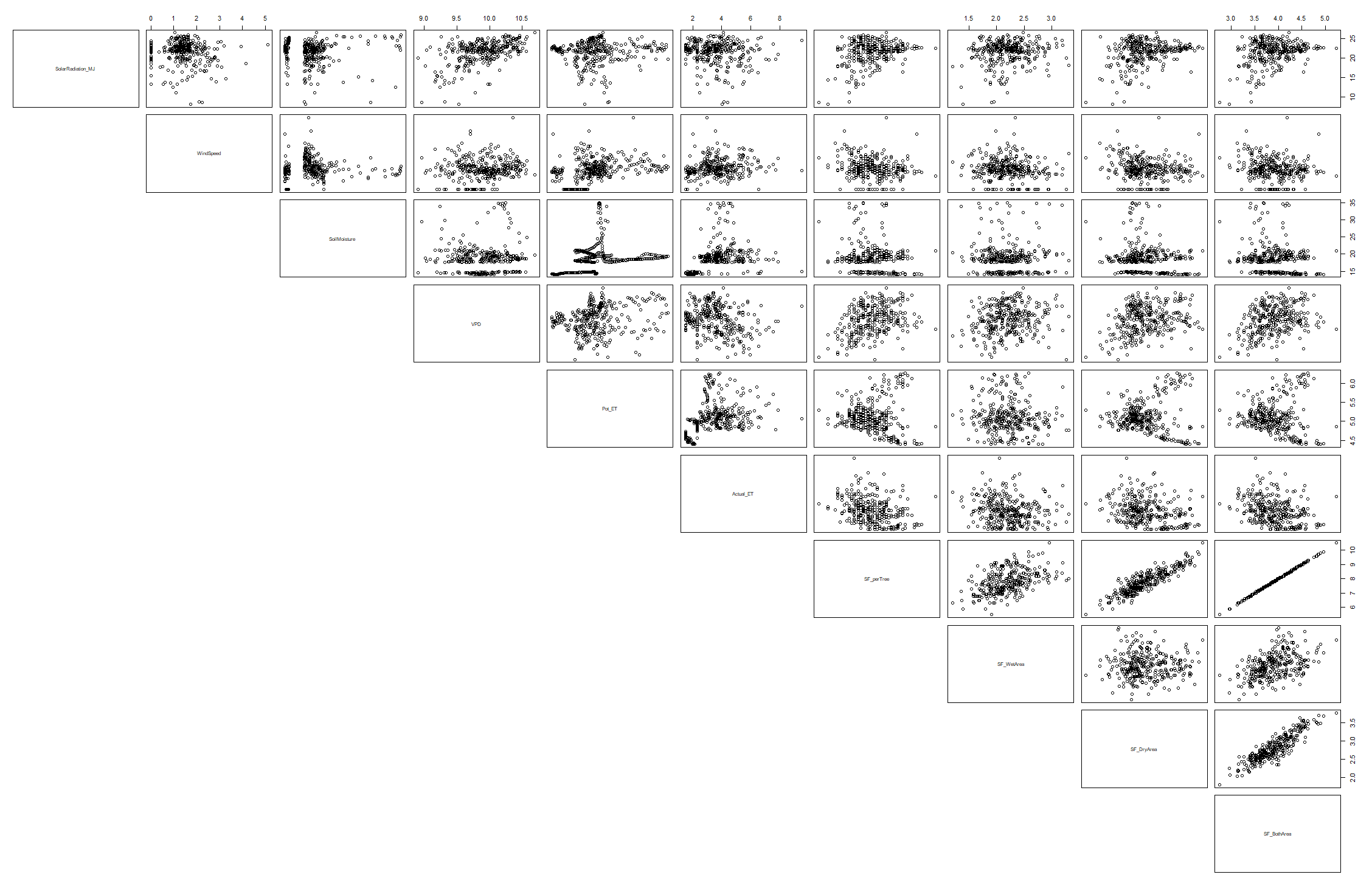

Supplement: Supplementary file 1 — Supplementary information [file 41598_2021_81776_MOESM1_ESM.docx]
